# Medication Use Patterns in Hospitalized Patients With COVID-19 in California During the Pandemic

**DOI:** 10.1001/jamanetworkopen.2021.10775

**Published:** 2021-05-21

**Authors:** Jonathan H. Watanabe, Jimmy Kwon, Bin Nan, Shira R. Abeles, Stanley Jia, Sanjay R. Mehta

**Affiliations:** 1 Department of Clinical Pharmacy Practice, University of California Irvine School of Pharmacy & Pharmaceutical Sciences, Irvine; 2 Department of Statistics, University of California, Irvine Donald Bren School of Information and Computer Sciences, Irvine; 3 Antimicrobial Stewardship, Infection Prevention and Clinical Epidemiology, Department of Medicine, University of California, San Diego School of Medicine, La Jolla; 4 Infectious Disease Section, VA San Diego Clinical Microbiology Laboratory, VA San Diego, La Jolla, California; 5 Department of Medicine, UC San Diego School of Medicine, La Jolla, California

## Abstract

This cohort study examines trends in medication use among patients hospitalized for COVID-19–related treatment in a large US university health care system from the start of stay-at-home orders in March 2020 throughout the rest of the year.

## Introduction

The novel SARS-CoV-2 virus has caused more than 118 million cases of COVID-19 and more than 2.6 million deaths worldwide.^1^ To evaluate the use of potential therapeutic options—including dexamethasone, remdesivir, enoxaparin, heparin, colchicine, hydrocortisone, tocilizumab, azithromycin, hydroxychloroquine, and medication classes of angiotensin-2 converting enzyme inhibitors (ACEIs) and angiotensin receptor blockers (ARBs)^2^—we measured daily and overall use percentages over the course of 2020 for hospitalized patients.

## Methods

This cohort study included patients confirmed positive by SARS-CoV-2 RNA detection from March 10, 2020, through December 31, 2020. Hospitalization within 30 days of diagnosis or test-confirmed positive with COVID-19 by SARS-CoV-2 RNA nucleic acid amplification with probe detection during stay was considered a COVID-19–related hospitalization. Data were from the University of California COVID Research Data Set (UC CORDS), which contains COVID-19 treatment information from all 5 UC Health medical centers (Davis, Irvine, Los Angeles, San Diego, and San Francisco). UC CORDS was operationalized by UC Health as non-human participant research and analyses are considered institutional review board exempt. The Strengthening the Reporting of Observational Studies in Epidemiology (STROBE) reporting guideline was followed.

Data were analyzed March 10, 2020, through December 31, 2020. Daily percentage utilization of medications was plotted based on diagnosis date fitting a nonparametric logistic regression model with time effect modeled by cubic splines in ggplot2 in R version 3.6.3 (R Project for Statistical Computing). Overall percentage use was also plotted over the pandemic. Statistical significance was set at α = .05 (eAppendix in the Supplement).

## Results

The total data set included 22 896 patients with COVID-19 (mean [SD] age, 42.4 [20.4] years; 12 154 [53.1%] women). Among the sample, 6326 patients (27.6%) were non-Hispanic White, 8475 (37.0%) were Hispanic, 1562 (6.8%) were Asian, and 1313 (5.7%) were Black. A COVID-19–related hospitalization occurred in 3546 patients (15.5%).

### Daily Use Analysis for Hospitalized Patients

**Figure 1.**
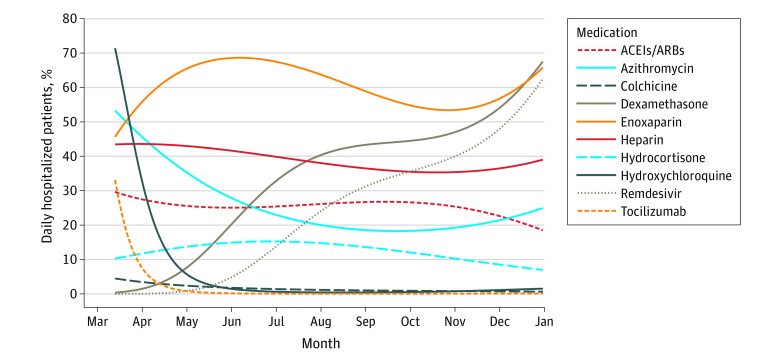
Daily Use Percentage of Potential Therapeutic Options Between March 2020 and January 2021 ACEI indicates angiontensin-2 converting enzyme inhibitor; and ARB, angiotensin receptor blocker.

Dexamethasone use increased from 1.4% (95% CI, 1.4%-1.5%) of diagnosed patients per day on March 31 to 67.5% (95% CI, 62.6%-72.1%) of patients per day on December 31 (Figure 1). Enoxaparin daily usage was 50.4% (95% CI, 45.7%-55.2%) on March 21 and remained above this percentage for the remainder of 2020. Remdesivir use increased more than 12-fold from 4.9% (95% CI, 4.7%-5.1%) on June 1 to 62.5% (95% CI, 56.7%-68.0%) on December 31. Azithromycin was used in 45.5% (95% CI, 41.9%-49.1%) on April 1, but fell to 20.0% (95% CI, 19.0%-21.1%) by August 1. Use of ACEIs/ARBs moderately declined from 27.5% (95% CI, 25.0%-30.2%) on March 31 to 18.5% (95% CI, 16.1%-21.1%) on December 31. Tocilizumab and colchicine uses were 2.4% (95% CI, 2.3%-2.5%) and 2.9% (95% CI, 2.8%-3.0%), respectively, on April 15 and remained below these percentages for the remainder of 2020.

### Overall Percentage Use in Hospitalized Patients Since March 2020

**Figure 2.**
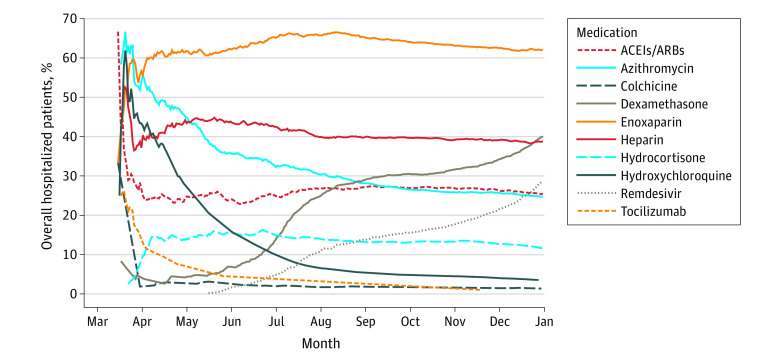
Overall Percentage of Hospitalized Patients Using Potential Therapeutic Option Between March 2020 and January 2021 ACEI indicates angiontensin-2 converting enzyme inhibitor; and ARB, angiotensin receptor blocker.

In early April 2020, more than 40% of patients up to that point (70 of 173 patients) had received hydroxychloroquine. By July, fewer than 10% of all patients (97 of 974 patients) had received it. Before May, dexamethasone was used in only 4% of all COVID-19 patients to that point (14 of 335 patients). However, use accelerated and by close of 2020 nearly 40% of all patients (1421 of 3546 patients) received dexamethasone. Heparin overall use was fairly stable over time, at approximately 40% (Figure 2).

## Discussion

This cohort study found that, early in the COVID-19 pandemic, antimicrobials azithromycin and hydroxychloroquine were each used in more than 40% of hospitalized patients. By June, use was below 30% and 5%, respectively. Enoxaparin use remained above 50% throughout 2020, perhaps because enoxaparin serves both for thrombosis prophylaxis and thrombophilia treatment triggered by COVID-19.^3^ Dexamethasone and remdesivir use grew substantially. One possible explanation is that remdesivir use may have corresponded with availability, as early in the pandemic it was predominantly available through trials in the UC system.^4^ Hydroxychloroquine use fell from over 40% to below 5% 2 months later. A small study conducted early in the pandemic favored use of hydroxychloroquine,^5^ but later, larger controlled studies found no benefit.^6^ While demographic characteristics of the study population were consistent with California, our findings are limited by their generalizability to the US. To our knowledge, this study represents the first analysis of medication utilization for hospitalized patients with COVID-19 in a large, diverse, statewide health system.

## Notes

Supplement. **eAppendix.** Statistical Analysis Summary
