# Supplementary material for: Medication Use Patterns in Hospitalized Patients With COVID-19 in California During the Pandemic
Source: JAMA Netw Open. 2021 May 21;4(5):e2110775. doi: 10.1001/jamanetworkopen.2021.10775 (PMC8140369; doi:10.1001/jamanetworkopen.2021.10775)
Supplement: Supplement. — eAppendix. Statistical Analysis Summary [file jamanetwopen-e2110775-s001.pdf]

Supplemental Online Content

Watanabe JH, Kwon J, Nan B, Abeles SR, Jia S, Mehta SR. Medication use patterns in hospitalized patients with COVID-19 in California during the pandemic. *JAMA Netw Open.* 2021;4(5):e2110775. doi:10.1001/jamanetworkopen.2021.10775

Supplement. eAppendix. Statistical Analysis Summary

This supplemental material has been provided by the authors to give readers additional information about their work.

© 2021 Watanabe JH et al. *JAMA Network Open.*

eAppendix. Statistical Analysis Summary

Daily percentage utilization of the potential therapeutic options in hospitalized patients was determined by modeling medication use based on day of diagnosis. Each curve is obtained from fitting a nonparametric logistic regression model with time effect modeled by cubic splines using five knots, where the three internal knots are placed at the 25th, 50th, and 75th percentiles of the time variable. and plotted using ggplot2 in R, version 3.6.3 (R Project for Statistical Computing). Overall percentage use was plotted for hospitalized patients over the study period by taking the total number of positive, hospitalized patients that had received the specific medication up to the month of interest divided by the total number of positive, hospitalized patients up to the same month of interest. Then plotting the overall monthly percentages. All analyses performed in R, version 3.6.3 (R Project for Statistical Computing). Statistical significance defined using two-sided significance level of alpha = 0.05.

© 2021 Watanabe JH et al. *JAMA Network Open.*
